# Brain reorganization: altered functional connectivity in reward network after stroke

**DOI:** 10.1016/j.nicl.2025.103914

**Published:** 2025-11-22

**Authors:** Yésica E. Martínez, Mario Widmer, Josua Zimmermann, Josef G. Schönhammer, Andreas R. Luft, Lutz Jäncke

**Affiliations:** a Department of Neuropsychology, University of Zurich, Zurich Switzerland; b Cereneo Center for Neurology and Rehabilitation, Vitznau, Switzerland; c Swiss Paraplegic Research, Nottwil, Switzerland; d Lake Lucerne Institute, Vitznau, Switzerland; e Department of Neurology, University Hospital of Zurich, Zurich, Switzerland; f Rehabilitation Engineering Laboratory, Department of Health Sciences and Technology, ETH Zurich, Zurich, Switzerland

**Keywords:** Stroke, fMRI, Reward network, Monetary reward, Motor skill learning

## Abstract

**Background:**

Motivation is key to functional recovery after stroke and is partly driven by reward pathway activation. Impaired reward-related striatal activation during motor learning may hinder rehabilitation that relies on motivational feedback. Even without direct damage to reward areas, stroke may disrupt reward network connectivity. What drives this reward processing network alterations is not known. This study examines how stroke alters reward-related functional connectivity during motor learning with monetary reward.

**Methods:**

Subacute stroke patients (*n* = 28) and age-matched healthy controls (*n* = 18) performed an arc-pointing motor learning task with the wrist and received performance-dependent monetary rewards during functional magnetic resonance imaging (fMRI). A generalized psychophysiological interactions model (gPPI) was used to evaluate differences in reward network functional connectivity.

**Results:**

In the reward interval, stroke patients exhibited significant functional connectivity changes within the reward network compared to healthy controls. Increased connectivity was observed between the ventral tegmental area, substantia nigra, striatum, precentral gyrus and orbitofrontal cortex, whereas decreased connectivity was found between ventral tegmental area and nucleus accumbens. Of note, motor performance and monetary reward did not differ between groups.

**Conclusion:**

Stroke leads to significant reward network reorganization, with both hyper- and hypoconnectivity in cortical and subcortical regions. Increased connectivity may reflect compensation; decreased connectivity may impair reward processing and learning. These findings support targeting reward pathways—e.g., via feedback or brain stimulation—to boost motor recovery.

## Highlights

• Stroke alters reward-related functional connectivity during motor learning with feedback.
• Hyperconnectivity observed between VTA, SN, striatum, OFC, and motor cortex after stroke.
• Hypoconnectivity noted between VTA and nucleus accumbens in stroke patients.
• Motor performance and received rewards were comparable between patients and controls.
• Reward network reorganization may inform strategies to enhance post-stroke recovery.

## 1 Introduction

Stroke remains a predominant cause of long-term disability worldwide, often resulting in impairments across motor, cognitive, and emotional domains (Langhorne, Bernhardt, Kwakkel, 2011). Functional recovery of stroke survivors is strongly influenced by motivation, which drives engagement in rehabilitation essential for improving functional outcomes (Rapolienė et al., 2018, Widmer et al., 2021). Performance feedback and rewards, such as monetary incentives, may enhance motivation and facilitating motor learning in rehabilitation. They were shown to accelerate motor skill acquisition, and to improve therapy participation (Malhotra et al., 2012, Vassiliadis et al., 2021, Wagner et al., 2023, Widmer et al., 2021).

Motivation is largely mediated by reward mechanisms linked to dopaminergic brain pathways (Haber, 2014). Stroke survivors often exhibit deficits in reward processing (Lam et al., 2016, Widmer et al., 2019, Wagner et al., 2023). Initial studies attributed these deficits to lesions in the basal ganglia, critical regions of the reward pathway essential for motor learning (Boyd et al., 2009, Dahms et al., 2020, Schmidt et al., 2008). This was further supported by studies showing impaired reward-related learning linked to striatal lesions (Lam et al., 2016, Malhotra et al., 2012), and degeneration of dopaminergic neurons in the substantia nigra following ischemic striatal infarction (Winter et al., 2015). However, studies indicate that impaired reward processing occurs regardless of whether the lesion impacts reward pathways (Widmer et al., 2019, Wagner et al., 2023). These findings suggest that affected reward processing in stroke patients likely stems from dysfunctions within reward circuitry, involving both subcortical and cortical regions, rather than lesional damage to a specific region of the reward pathway (Haber & Knutson, 2010; Widmer et al., 2019, Wagner et al., 2023). This highlights a broader network dysfunction, indicating that besides localized brain damage stroke also affects network-level reward processing mechanisms.

The brains’ reward network comprises key structures such as the ventral tegmental area (VTA) and the substantia nigra (SN), both with dopaminergic projections to the ventral striatum (VS), which includes the nucleus accumbens (NAcc), as well as the ventral caudate and putamen (Leemburg et al., 2018, Haber, 2014; Haber & Knutson, 2010). The VS directly innervates the VTA and SN, and indirectly connects to the pallidum, which subsequently also communicates back to the VTA and SN, forming a feedback loop (Li et al., 2015). This complex interconnected loop plays a crucial role in modulating dopamine release and reward processing, integrating emotional, cognitive, and motor functions (Haber, 2014). Furthermore, bidirectional signaling occurs between frontal areas (e.g., orbitofrontal cortex) and the VS. The dorsal striatum (caudate and putamen) also receives projections from these frontal regions (Haber, 2014). The primary motor cortex receives dopaminergic input from the VTA (Hosp et al., 2011). These projections are essential for motor learning (Hosp et al. 2011; Molina-Luna et al., 2009).

Performance-based rewards in motor tasks significantly enhance activity in this circuit, highlighting the importance of dopaminergic pathways in motor learning in mice (Hosp et al., 2011, Schott et al., 2008) and humans (Lutz et al., 2012, Widmer et al., 2016). Specifically, dopamine facilitates synaptic plasticity within the precentral gyrus and striatum, enabling the consolidation of motor memories and promoting efficient neural communication essential for motor skill learning (Hosp et al., 2011; Molina-Luna et al., 2009; Lutz et al., 2012). Once a motor skill is learned, however, its execution does not rely on the VTA-to-M1 dopaminergic projection (Hosp et al., 2011).

Taken together, recent evidence suggests that impairments in reward processing after stroke cannot be fully explained by focal lesions to single brain structures, such as the basal ganglia or dopaminergic midbrain nuclei. Instead, these deficits appear to reflect broader dysfunction within the reward network—a distributed set of interconnected regions spanning cortical and subcortical nodes—including the ventral striatum, prefrontal cortex, and midbrain structures (Widmer et al., 2019; Haber & Knutson, 2010; Wagner et al., 2023). Similarly, network neuroscience proposes that stroke disrupts not only localized brain areas but also the functional integration between regions necessary for complex cognitive and motivational processes (Carrera & Tononi, 2014). This supports the hypothesis that alterations in the functional connectivity of the reward network may be associated with impaired motivational drive and reward responsiveness observed in stroke survivors.

To test this hypothesis, we measured functional connectivity which refers to the statistical interdependencies of functional magnetic resonance imaging (fMRI) time series between distinct brain regions during rest or specific tasks, and is used to assess how functional networks, such as the reward network, operate in synchrony. Unlike the conventional analysis approach, which examines activation levels within single regions of interest (ROIs) without considering their correlations with other regions (Widmer et al., 2019), functional connectivity offers insight into network-level activation patters. More precisely, we assessed functional connectivity using a generalized psychophysiological interaction (gPPI) analysis, a regression-based extension of the conventional PPI approach. Unlike univariate fMRI analyses that focus solely on regional activation amplitudes, gPPI models context-dependent changes in the correlation of activity between brain regions across different psychological conditions (McLaren et al., 2012). This method is particularly suited for task-based paradigms involving multiple intervals, such as reward versus no-reward trials, allowing for a fine-grained examination of how specific task contexts modulate functional coupling between brain areas. Compared to simple seed-based connectivity or classical PPI, gPPI provides increased sensitivity and flexibility in modeling network dynamics, making it ideal for identifying functional connectivity alterations within the reward network during monetary reward processing.

Participants performed a motor task with performance-dependent monetary reward, designed to activate the reward network through contingent feedback and monetary reward. We hypothesized that stroke survivors would exhibit altered functional connectivity between critical nodes of the reward network compared to healthy controls during monetary reward processing. Specifically, based on previous findings of impaired reward responsiveness and dopamine dysregulation after stroke (Lam et al., 2016, Widmer et al., 2019, Wagner et al., 2023), we expected to observe reduced task-related coupling between the ventral striatum and prefrontal areas (e.g., orbitofrontal cortex), as well as between the ventral striatum and motor regions. These changes would reflect impaired integration of motivational signals into motor planning and execution, potentially contributing to reduced engagement and learning during rehabilitation. At the same time, based on previous literature, we could also expect opposite patterns such as hyperconnectivity within reward–motor pathways, reflecting adaptive or maladaptive network reorganization processes following neural disruption (Hummel and Cohen, 2005, Aswendt and Hoehn, 2023).

## 2 Material and methods

### 2.1 Participants

In total, *n* = 34 subacute stroke survivors (*n* = 28 included in the analysis, *n* = 6 excluded for incomplete imaging data) and *n* = 18 age-matched healthy adults participated in the study. Stroke patients were recruited at a local hospital and a local rehabilitation clinic. Stroke patients were excluded if they had serious conditions such as aphasia, dementia, or pre-existing depression. Control participants were excluded for psychiatric disorders or the use of central nervous system medications, such as antidepressants. General exclusion criteria included MRI safety contraindications and uncorrectable visual impairments. The use of antidepressants was not an exclusion criterion for stroke patients due to their common use in the subacute stroke phase. All participants were unfamiliar with the task, received identical instructions, and underwent identical study procedures. Participants received financial compensation according to their performance in the motor task, and the amount received was comparable across all participants. Data were collected between November 2014 and January 2019. The study was approved by the local ethics committee (EKNZ BASEC 2016–00,079), and written informed consent was obtained from all participants in accordance with the Declaration of Helsinki.

### 2.2 Study procedure

The study procedure and motor task were identical to Widmer et al., (2019), with the data on univariate task activation patterns previously published in Widmer et al. (2019). In brief, participants performed an arc-pointing task, an upper-limb motor skill learning task, during fMRI. The motor task included an interval with feedback plus performance-dependent monetary reward, and a control interval with no-feedback and no-reward. The study was conducted in a single measurement session at the cereneo Center for Neurology and Rehabilitation in Vitznau, Switzerland. After providing informed consent, participants completed the *Beck Depression Inventory* (BDI II; Beck et al., 1961), *Montreal Cognitive Assessment* (MoCA; Nasreddine et al., 2005), and *Edinburgh Handedness Inventory* (EHI; Williams, 1986) for screening. From patient records, we additionally extracted clinical measures including the National Institutes of Health Stroke Scale (NIHSS; Brott et al., 1989), the Fugl-Meyer Assessment (FMA, Fugl-Meyer et al., 1975), and information on antidepressant use (yes/no). Upon completing the fMRI task, participants filled out the *Intrinsic Motivation Inventory* (IMI, Ryan, 1982). Lastly, participants gave subjective rating of monetary reward value on a 7-point Likert scale.

### 2.3 Motor task

To engage reward-related motor processing, participants performed the arc-pointing task during fMRI (cf. Shmuelof et al., 2012; Widmer et al., 2016, 2017), earning monetary rewards based on performance. Stroke patients used the unaffected hand; controls used their dominant hand. Cursor movement, controlled via wrist motions tracked by an MRI-compatible motion capture system (Oqus MRI, Qualysis AB, Gothenburg, Sweden), guided a marker through a semicircular arc. A familiarization phase calibrated task difficulty to equalize reward likelihood (∼50 % reward trials) across individuals. During scanning, participants completed four blocks of 25 trials at fixed difficulty. Both the duration of cursor placement and the movement time were self-paced, resulting in variable trial lengths. Feedback indicated whether performance exceeded prior median accuracy: reward trials showed earned CHF and trajectory (“FB + Reward”); no-reward trials showed neutral symbols only (“NoFB + NoReward”). When the performance in the current trial was better than previous trials (reward trials), participants were shown the amount earned in Swiss Francs (CHF) (e.g., “In diesem Versuch gewonnen: 0.7 CHF”) and the cumulative earnings (e.g., “Total: 0.7 CHF”). Additionally, their cursor trajectory was displayed for visual performance feedback (“FB + Reward”). When the performance in the current trial was worse than in previous trials (no-reward trials), question marks (neutral stimuli) replaced the monetary feedback, and no cursor trajectory was shown (“NoFB + NoReward”). Performance was measured as the percentage of the cursor trajectory within the arc ribbon, which directly determined the reward value. For instance, if 80 % of the trajectory was within the arc ribbon and exceeded the median of the previous 10 trials, participants earned 0.80 CHF (≈ 0.80 USD).

### 2.4 fMRI data acquisition and processing

The fMRI data was acquired on a Philips Ingenia 3.0 T MRI scanner (Philips Healthcare, Best, The Netherlands) with a 32-channel head coil. Prior to the fMRI session, a T1-weighted 3D magnetization-prepared rapid gradient-echo (MPRAGE) sequence was run. The MPRAGE sequence comprised 170 slices, repetition time (TR) = 6.8 ms, echo time (TE) = 3.1 ms, flip angle = 8°, field of view (FOV) = 256 x 240 x 204 mm, matrix size = 256 × 240, and voxel size = 1 x 1 x 1.20 mm. Subsequently, fMRI data was collected employing a sensitivity-encoded (SENSE, factor 1.8) single-shot echo planar imaging technique (EPI) with a TR = 2.35 s, TE = 32 ms, FOV = 240 x 240 x 140 mm, flip angle = 82°, matrix size = 80 × 80, and voxel size = 3 x 3 x 3.5 mm. Each run comprised 350 volumes, yielding a total acquisition time of approximately 13 min and 42 s. Three dummy scans were acquired to achieve steady state magnetization. fMRI data were preprocessed and analyzed using CONN (functional Connectivity Toolbox) (Whitfield-Gabrieli & Nieto-Castanon, 2012) in Matlab R2023b, requiring SPM12 packages (Statistical Parametric Mapping, Institute of Neurology, London, UK; http://www.fil.ion.ucl.ac.uk/sp). The preprocessing pipeline included motion and slice-timing correction, identification of motion outlier volumes (Artifact Detection Tool in CONN, based on framewise displacement > 0.9 mm and global signal changes > 5 standard deviations (Nieto-Castanon, 2020)), linear registration to T1 as well as non-linear normalization to MNI space, smoothing with a 4 mm full-width at half-maximum (FWHM) Gaussian kernel. Then data were denoised by regressing out potential confounding effects in the BOLD signal (Power et al., 2014), which included head motion parameters (translation, rotation, and their first-order derivatives), motion outlier volumes, physiological noise components from segmented white matter and cerebrospinal fluid using aCompCor (Behzadi et al., 2007), session-specific trends or task-related effects (Whitfield-Gabrieli & Nieto-Castanon, 2012), and by additionally applying a high-pass filter of 0.008 Hz. Normalization and ROI localization were visually inspected for each subject to ensure accurate alignment, with particular attention to small regions such as the VTA. To illustrate the quality of normalization and ROI localization, three representative examples are provided in the supplementary material (Supplementary Figs. S1 and S2).

### 2.5 Regions of interest

ROIs were selected based on previous literature research on (monetary) reward processing (Haber, 2014; Haber & Knutson 2010; Liu et al., 2011, Sescousse et al., 2013, Schmidt et al., 2008). We defined the following seven regions as the reward network: VTA, SN, NAcc, Pallidum, Caudate, Putamen, FOrb, Precentral gyrus. ROI masks from the Harvard-Oxford Cortical and Subcortical Structural Atlases (Kennedy et al., 2016), as included in the CONN toolbox, were used. For the VTA and SN ROIs, masks from Pauli, Nili, & Tyszka (2018) were utilized.

### 2.6 General psychophysiological interactions analysis

As input to the ROI-to-ROI functional connectivity analysis in CONN, we used preprocessed BOLD time-series data from each voxel within predefined ROIs. For the ROI-to-ROI first-level functional connectivity analysis in CONN, we used a univariate regression gPPI model producing beta weights (McLaren et al., 2012). This approach differs from traditional functional connectivity methods, which provide an averaged measure of connectivity over time. In gPPI, the activity in the target ROI is explained by the activity in the seed ROI, the task intervals, and their interaction (McLaren et al., 2012). This approach was chosen because our participants received feedback and reward in an event-related design (reward feedback = 3 s), making it essential to capture task-modulated changes in connectivity with temporal specificity. We modeled the connectivity between a seed region and a target region by focusing on the reward/no-reward intervals (“FB + Reward”, “NoFB + NoReward”) as the task intervals of interest. These contrasts inherently conflate reward and feedback, which is an accepted trade-off in this paradigm since reward information is conveyed through the feedback signal. All other intervals (“move to start,” “wait for start,” “movement phase,” and “wait for feedback”) were not explicitly modeled as conditions of interest and therefore served as baseline. In this context, implicit baseline refers to the average connectivity across these remaining task phases, which provides the reference against which the specific beta weights of interest are estimated. As output, the first-level gPPI analysis yielded condition-specific interaction beta weights, which represent the regression coefficients of task-dependent functional connectivity between each seed-target ROI pair during the task intervals of interest relative to this implicit baseline. At the second level, we performed two-sample two-sided *t*-tests to compare connectivity between stroke survivors and healthy controls with a threshold connection of *p* < 0.05. We controlled for age, sex, and BDI scores. The reported effect sizes reflect the condition-specific interaction beta weights of each participant. All second-level ROI-to-ROI functional connectivity results were corrected for multiple comparisons using a false discovery rate (FDR) of *p < 0.05* at cluster level. Clusters were defined by CONN’s hierarchical clustering of ROIs based on functional similarity, with all edges within and between network pairs grouped into clusters. Cluster-level significance was assessed using a multivariate omnibus F-test, with FDR correction (FDR-corrected *p* < 0.05) applied across the full set of clusters (Whitfield-Gabrieli & Nieto-Castanon, 2012). In addition, individual ROI-to-ROI connections within significant clusters were also reported with their own FDR-corrected *p*-values.

Further, we conducted sensitivity analyses to examine the robustness of our results. First, we included antidepressant use as an additional covariate, given its potential influence on reward-related networks. Second, we added MoCA scores as a covariate to account for group differences in global cognition. As an exploratory step, we also tested whether time since stroke (in days) was associated with task-dependent connectivity within the stroke group (i.e., main effect of time since stroke). Cluster-level FDR correction was applied as in the main ROI-to-ROI analyses. Lastly, as a robustness check, we also repeated the second-level comparisons in five randomly drawn, balanced subsamples (18 S vs. 18 controls) to ensure that the observed effects were not driven by unequal sample sizes between groups.

### 2.7 Overlap of stroke lesions and ROIs

Stroke lesions were manually segmented on the FLAIR image in native space and normalized to MNI space. Overlap of the stroke lesion with each ROI was computed as percentage.

### 2.8 Statistical analysis of behavioral data

We tested for group differences in behavioral data using Mann-Whitney U tests (Wilcoxon rank-sum tests) for motor performance (% in channel, and movement time), MoCA scores, BDI scores, IMI scores, and age. The Mann-Whitney *U* test is a non-parametric alternative to the independent samples *t*-test and is appropriate for comparing two independent groups when normality assumptions are violated. For the categorical variable sex, we used a Chi-squared test of independence. Effect sizes (r) are calculated as: r = Z / sqrt(N), where Z is the standard normal approximation of U, and N is the total sample size (Rosenthal, 1991). Effect sizes (Rosenthal’s r) were categorized as small (.10 ≤ r < 0.30), medium (.30 ≤ r < 0.50), or large (r ≥ 0.50), following Cohen's guidelines (1988).

To account for potential non-independence of measurements between groups, we conducted two generalized linear mixed models (GLMMs) using a Gamma distribution with a log link function to accommodate the non-normal distribution of the dependent variables. The first model investigated the relationship between the % in channel and the predictors block, group, and their interaction. The second model explored the relationship between movement time and the same predictors. Both models included random intercepts for each participant to account for repeated measures within participants. Intraclass correlation coefficients (ICC) were calculated to evaluate the degree of non-independence within participants for both models. Effect sizes are reflected by standardized beta weights. To control for multiple comparisons, False Discovery Rate (FDR) correction (Benjamini & Hochberg, 1995) was applied to all *p*-values. Statistical analysis of behavioral data was performed using R version 4.5.0.

## 3 Results

### 3.1 Behavioral results

Basic behavioral results reported here have already been published in Widmer et al. (2019). For contextual understanding, we briefly summarize the key findings relevant to the current study. Additionally, we provide more comprehensive analysis of behavioral measures, including new results of motor performance dynamics. We excluded six stroke patients due to incomplete data (n = 2 FLAIR and n = 4 T2 images missing). Of these, two patients were unable to complete the main part of the experiment due to technical issues, while another two had to stop prematurely (one due to claustrophobia and the other because of fatigue). Additionally, one control subject was excluded due to the intake of medication affecting the central nervous system. Table 1 presents the demographic, clinical, and behavioral characteristics of the analyzed sample of 28 S patients and 18 healthy controls. Regarding clinical variables, 15 of 28 S patients (54 %) reported current antidepressant use, 12 patients (43 %) reported no use, and information was missing for 1 patient, whereas none of the controls reported antidepressant use. The mean time since stroke was 52.32 ± 24.24 days (range 25–127 days). Stroke severity as measured by the NIHSS (*n* = 14) indicated relatively mild to moderate deficits (*M* = 5.93 ± 5.23). NIHSS was not available for other patients. Upper-extremity motor impairment as assessed with the Fugl–Meyer Assessment (FMA; *n* = 26) indicated mild to moderate impairment (*M* = 46.04 ± 16.53). Two patients were missing FMA data because the measure had not been documented by the local physiotherapists.

**Table 1:** Characteristics of the sample.

	Stroke patients (*n* = 28)	Controls (*n* = 18)	Test statistic	*p*	*Effect size r*
Age	60.32 (±13.55)	65.39 (±6.40)	*W* = 331.5	0.075	0.26
Sex (female/male)	8/20	6/12	*χ^2^* = 0.0	0.989	−
Handedness (right/left/bimanual)	24/2/2	15/0/3	−	−	−
BDI II	8.04 (±6.09)*	1.78 (±1.93)	*W* = 56.5	0.000	0.65
Antidepressant use (yes/no/missing)	15/12/1	0/18/0	−	−	−
Time since stroke (days)	52.321 (±24.241)	−	−	−	−
NIHSS (*n* = 14)	5.93 (±5.23)	−	−	−	−
MoCA	23.71 (±5.15)*	27.39 (±2.09)	*W* = 396.5	0.001	0.48
FMA, *n* = 26)	46.04 (±16.53)				
IMI	4.57 (±.77)	4.71 (±.57)	*W* = 94.0	0.979	0.02
PCTin (%)	55.38 (±14.57)	54.03 (±10.13)	*W* = 248.0	0.937	0.01
Movement time (s)	6.77 (±2.10)*	4.42 (±1.20)	*W* = 78.0	0.000	0.58
*Note:* Values reflect count or mean ± standard deviation or number per category. Significant differences between groups are indicated by * (*p* < 0.05). BDI = Beck Depression Inventory; range of time since stroke: 25–127 days; NIHSS = National Institutes of Health Stroke Scale; MoCA = Montreal Cognitive Assessment, FMA = Fugl-Meyer Assessment; PCTin = Percentage in Channel, s = seconds. Lesion side and lesion volume are described elsewhere in the manuscript (Fig. 2, Table 3).					

The results revealed a significantly larger MoCA score for controls than stroke patients (*M* controls = 27.39 ± 2.09 points, *M* stroke patients = 23.71 ± 5.15 points, *W* = 396.5, *Z* = 3.27, *p* = 0.001, *r* = 0.48). Similarly, stroke patients had significantly higher BDI scores (*M* controls: 1.78 (±1.93) points, *M* stroke patients: 8.04 (±6.09) points, *W* = 56.5, *Z* = 4.41, *p* = 0.000, *r* = 0.65). A significant difference was also observed for mean movement time, whereby stroke patients were slower (*M* controls: 4.42 (±1.20) seconds, *M* stroke patients: 6.77 (±2.10) seconds, *W* = 78.0, *Z* = 3.91, *p* = 0.000, *r* = 0.58). However, no significant differences were found for mean % in channel, (*W* = 248.0, *Z* = 0.08, *p* = 0.937, *r* = 0.01), age (*W* = 331.5, *Z* = 1.78, *p* = 0.075 *r* = 0.26), or sex, *χ^2^* = 0.00, p = 0.989. Additionally, intrinsic motivation (IMI *M* stroke = 4.57 ± 0.77, *M* controls = 4.71 ± 0.57) and the subjective rating of monetary value (*M* stroke = 2.44 ± 0.942, *M* controls = 2.83 ± 1.24) together, did not differ significantly between groups (*W* = 94.0, *Z* = 0.16, *p* = 0.869, *p* = 0.979, *r* = 0.02). For the IMI and the subjective rating of monetary value, data were available for only *n* = 20 S patients and *n* = 9 healthy controls, as these questionnaires were added after the study had already started. Taken together, these results suggest that MoCA, BDI, and movement time differed significantly between groups, while percentage in channel, age, sex, and intrinsic motivation did not show significant group differences.

**Fig. 1:**
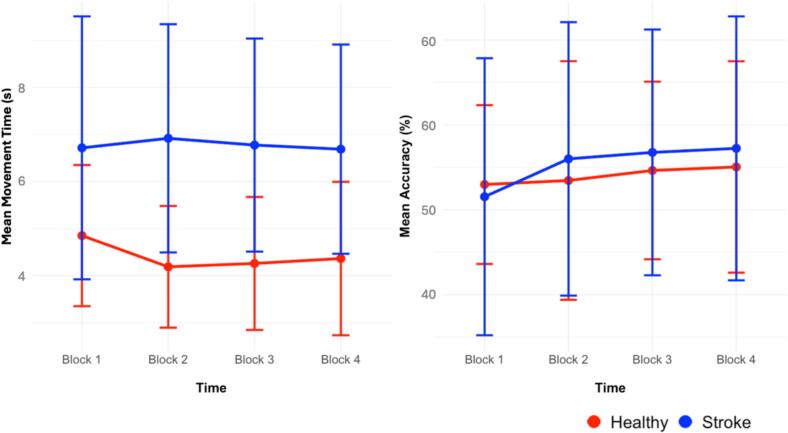
Mean movement time over time (in seconds) and accuracy over time (in %). Points reflect mean and error bars standard deviation. Higher mean movement values in seconds indicate slower movement execution.

Further analysis revealed that measurements of percentage in channel were moderately non-independent within participants (*ICC*(1) = 0.54, *F*(1, 182) = 14.30, *p* < 0.001), indicating highly reliable group-level differences (*ICC*(2) = 0.98). A GLMM with a Gamma family and log link was conducted to investigate the effects of time and group on percentage in channel while accounting for random intercepts for each participant. There was no significant difference between group and between blocks (see Table 2). The random intercept variance for participants was *σ2* = 0.025, and residual variance was *σ2* = 0.022. Measurements of movement time were moderately non-independent within participants (*ICC*(1) = 0.47, *F*(1, 182) = 11.44, p < 0.001), indicating highly reliable group-level differences (*ICC*(2) = 0.98). We found a significant main effect of group, indicating that the stroke group had a slower performance (i.e., higher response times in seconds) compared to the healthy group (*β* = 0.300, *SE* = 0.133, *z* = 2.26, *p* = 0.024, *p_FDR_* = 0.088). Further, movement time increased numerically in block 2 (*β* = -0.144, *SE* = 0.064, *z* = -2.24, *p* = 0.025, *p_FDR_* = 0.088) and block 3 (*β* = -0.142, *SE* = 0.065, z = -2.21, *p* = 0.027, *p_FDR_* = 0.088). After FDR correction, no significant effects remained (*p_FDR_* > 0.05, see Table 2). Random intercept variance for participants was *σ2* = 0.211, and residual variance was *σ^2^* = 0.226. Fig. 1 presents the mean movement time and mean % of time in channel across training. Both groups demonstrated comparable learning trajectories, as indicated by the convergence of their performance curves. Overall, the observed trend of decreasing response times (i.e., faster performance) and increasing accuracy across blocks suggests motor learning in both patients and controls.

**Table 2:** Behavioral effects with FDR adjustment.

**Dependent Variable**	**Effect**	***β***	***Std. Error***	***z-value***	***p-value***	***p_FDR_***
**% in Channel**	(Intercept)	−5.237	0.079	−66.14	< 0.001	< 0.001
	Block 2	−0.005	0.039	−0.13	0.893	0.893
	Block 3	0.027	0.039	0.69	0.490	0.558
	Block 4	0.029	0.039	0.74	0.459	0.558
	Group	−0.057	0.101	−0.56	0.576	0.614
	Block 2 × Group	0.086	0.050	1.71	0.087	0.155
	Block 3 × Group	0.078	0.050	1.56	0.119	0.159
	Block 4 × Group	0.080	0.050	1.61	0.108	0.158
**Movement Time**	(Intercept)	1.539	0.104	14.86	< 0.001	< 0.001
	Block 2	−0.144	0.064	−2.24	0.025	0.088
	Block 3	−0.142	0.065	−2.21	0.027	0.088
	Block 4	−0.123	0.065	−1.91	0.056	0.112
	Group	0.300	0.133	2.26	0.024	0.088
	Block 2 × Group	0.174	0.082	2.11	0.034	0.092
	Block 3 × Group	0.162	0.083	1.95	0.051	0.112
	Block 4 × Group	0.136	0.083	1.64	0.100	0.158

### 3.2 Overlap of stroke lesion with ROIs

**Fig. 2:**
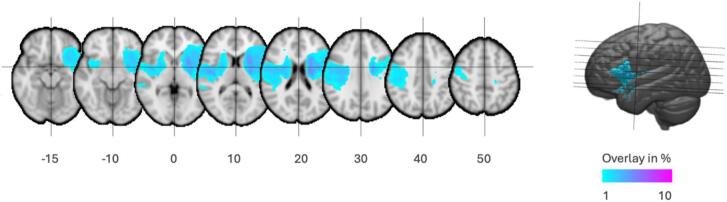
Lesion overlap map across all subjects. Overlay of individual lesion masks in MNI space, projected onto axial slices of the MNI152 template. Color intensity indicates the percentage of patients with lesions at each voxel, ranging from 1% (blue) to 10% (purple), as illustrated in the color bar. Lesion overlap was generally low, with a maximum voxel-wise overlap of 7% across stroke participants. Right hemisphere is displayed on the left side of the image. (For interpretation of the references to colour in this figure legend, the reader is referred to the web version of this article.)

Overall, lesions were rather small (mean lesion volume = 22.75 ± 40.37 mL, maximum = 188.98 mL). Lesion overlap across all subjects was low (see Fig. 2), with a maximum voxel-wise overlap of 7 %. Stroke lesions of patients only minimally overlapped with the used ROIs of the reward network, with all ROIs overlapping 6 % at most with lesion on average across all patients, except the left putamen ROI with 17 % overlap on average (see Table 3). However, two patients accounted for the largest lesion overlap with 23 % (mostly left caudate, putamen and pallidum) and 19 % (left caudate and pallidum), respectively.

**Table 3:** Lesion overlap in % averaged over all stroke patients.

**ROI**	**Left (%)**	**Right (%)**
**Pallidum**	6	2
**Caudate**	6	1
**Putamen**	17	4
**Forb**	2	0
**PreCG**	0	1
**NAcc**	2	0
**VTA**	0	0
**SN**	0	0
*Note*: Forb = orbitofrontal cortex, PreCG = precentral gyrus, NAcc = nucleus accumbens, VTA = ventral tegmental area, SN = substantia nigra.		

### 3.3 Functional connectivity results

**Fig. 3:**
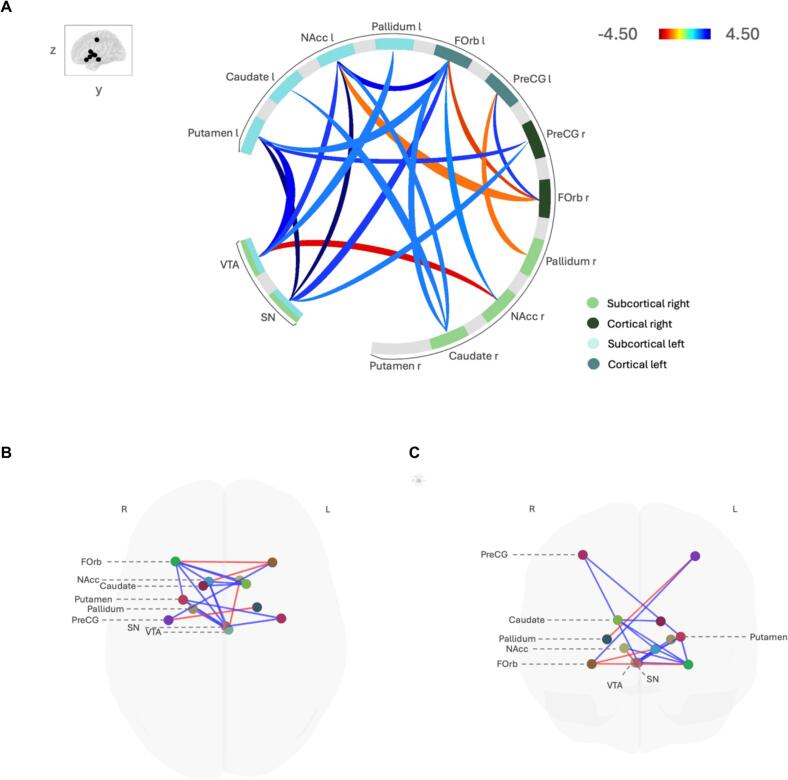
*(*A) Wheel representation of the two significant clusters of ROI-to-ROI functional connectivity within the reward network for stroke patients compared to healthy controls (stroke > healthy controls) during the reward interval (*p_FDR_* < 0.05). Color bars represent *t*-values. Red lines indicate lower functional connectivity, and blue lines indicate higher functional connectivity in stroke patients relative to healthy controls. (B) Superior view illustrating significant differences between stroke patients and healthy participants, where red lines indicate lower functional connectivity and blue lines indicate higher functional connectivity in stroke patients relative to healthy controls. (C) Anterior view of the same connectivity differences. Stroke patients exhibited both increased and decreased functional connectivity within key regions of the reward network. VTA = ventral tegmental area, SN = substantia nigra, NAcc = nucleus accumbens, FOrb = orbitofrontal cortex, PreGC = precentral gyrus. (For interpretation of the references to colour in this figure legend, the reader is referred to the web version of this article.)

**Fig. 4:**
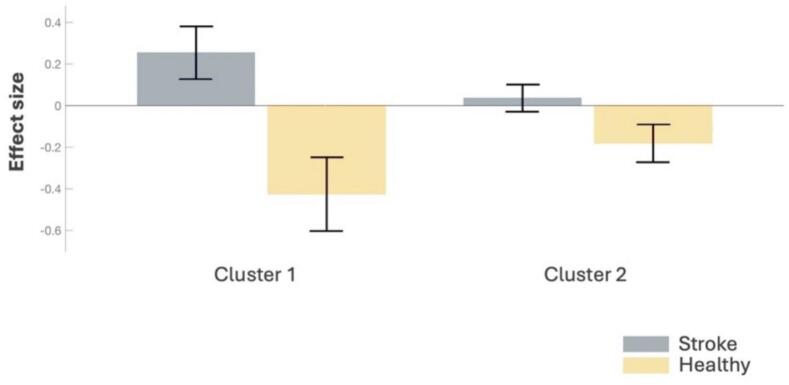
Effect sizes (*β*-weights). Effect sizes (*β*-weights) across significant ROI-to-ROI functional connections within the reward network for stroke patients compared to healthy controls during the FB + Reward interval (*p_FDR_* < 0.05).

A regression gPPI analysis was conducted to examine group differences in functional connectivity between stroke patients and healthy controls, while controlling for age, sex, and depression score (BDI). Two significant clusters were identified (see Table 4, Fig. 3, Fig. 4), revealing distinct patterns of functional connectivity in the reward pathway. Cluster 1 revealed significant functional connectivity changes (*F*(2,40) = 6.10, *p* = 0.005, *p_FDR_* = 0.015) primarily involving the SN, NAcc, Putamen, and VTA. Effect sizes are represented by the standardized beta weights (*β*) derived from the gPPI regression model, indicating the strength of task-modulated connectivity between regions (see Table 4). Increased functional connectivity was observed between the left Putamen and SN (*t* = -4.50, *p_FDR_* = 0.001), the SN and left NAcc (*t* = -4.18, *p_FDR_* = 0.001), the SN and left Putamen (*t* = -4.11, *p_FDR_* = 0.001), the left NAcc and SN (*t* = -3.52, *p_FDR_* = 0.014), and the left Putamen and VTA (*t* = -3.13, *p_FDR_* = 0.021). Additionally, increased connectivity was detected between the SN and left FOrb (*t* = -2.64, *p_FDR_* = 0.050). In contrast, decreased functional connectivity was observed between the right NAcc and VTA (*t* = 3.28, *p_FDR_* = 0.027). Cluster 2 demonstrated altered functional connectivity (*F*(2,40) = 4.04, *p_FDR_* = 0.038) in the NAcc, FOrb, Putamen, and Precentral gyrus. Specifically, increased functional connectivity was identified between the left NAcc and left FOrb (*t* = -3.27, *p_FDR_* = 0.014) and between the left Putamen and right PreGC (*t* = -2.78, *p_FDR_* = 0.036). Overall, the cluster-level FDR correction indicates reliable group differences in task-dependent connectivity. Several individual ROI-to-ROI connections reached uncorrected significance but did not survive connection-level FDR correction. Importantly, in contrast, no significant group differences were observed in the reward network during the “NoFB + NoReward” interval (*p_FDR_* > 0.05).

**Table 4:** Significant clusters of activation identified with the stroke > healthy contrast in the “FB + reward” interval.

**Cluster**	**Statistics F(2,40)**	**Connection**	***β-weight***	***T*-value**	***p_FDR_***	**FC in Stroke**
**1**	**6.10**			**−**	**0.015**	**−**
		**Putamen l – SN**	**2.05**	**4.5**	**0.001**	**Higher**
		**SN – NAcc l**	**0.38**	**4.18**	**0.001**	**Higher**
		**SN – Putamen l**	**0.20**	**4.11**	**0.001**	**Higher**
		**NAcc l – SN**	**0.90**	**3.52**	**0.014**	**Higher**
		**Putamen l – VTA**	**2.07**	**3.13**	**0.021**	**Higher**
		**NAcc r – VTA**	**−0.90**	−**3.28**	**0.027**	**Lower**
		**SN – FOrb l**	**0.16**	**2.64**	**0.05**	**Higher**
		NAcc l **–** VTA	0.71	2.57	0.06	Higher
		VTA **–** Putamen l	0.11	2.86	0.086	Higher
		VTA **–** NAcc l	0.24	2.56	0.093	Higher
		Precentral gyrus r **–** SN	1.23	2.2	0.216	Higher
		Pallidum l **–** VTA	1.00	2.26	0.382	Higher
**2**	**4.04**			**−**	**0.038**	**−**
		**NAcc l – FOrb l**	**0.28**	**3.27**	**0.014**	**Higher**
		**Putamen l – Precentral gyrus r**	**0.21**	**2.78**	**0.036**	**Higher**
		FOrb l **–** FOrb r	**−0**.38	**−**2.87	0.085	Lower
		Putamen l **–** FOrb l	0.44	2.25	0.097	Higher
		FOrb r **–** Precentral gyrus l	0.38	2.77	0.11	Higher
		FOrb r **–** NAcc l	**−0**.71	**−**2.39	0.139	Lower
		FOrb l **–** NAcc l	0.80	2.34	0.159	Higher
		FOrb l **–** Caudate r	0.47	2.08	0.165	Higher
		NAcc r **–** FOrb l	0.20	2.26	0.192	Higher
		Precentral gyrus r **–** Putamen l	0.49	2.24	0.216	Higher
		Caudate r **–** NAcc l	0.42	2.17	0.255	Higher
		Caudate r **–** Caudate l	0.27	2.13	0.255	Higher
		Precentral gyrus l **–** FOrb r	0.45	2.36	0.301	Higher
		Pallidum r **–** Precentral gyrus l	**−0**.19	**−**2.22	0.413	Lower

*Note:* FC = Functional Connectivity, *β*-weight representing the interaction between seed ROI activity and task interval (“FB + reward”), as modeled in gPPI analysis. SN = substantia nigra, NAcc = nucleus accumbens, VTA = ventral tegmental area, Forb = orbitofrontal cortex, PreCG = precentral gyrus. For robustness under balanced group sizes, see Supplementary Table S3a,b (five random 18-vs-18 runs).

As sensitivity analyses, we first included antidepressant use as an additional covariate in the gPPI models. The core findings were preserved (e.g., increased connectivity in stroke for Putamen–SN and SN–NAcc, and decreased connectivity for NAcc–VTA), although some individual connections were attenuated and did not remain significant after FDR correction, particularly those involving FOrb and PreCG (see Supplementary Table S1 for a detailed list of replicated connections). We then added MoCA as a covariate. While the overall pattern and direction of effects were preserved, fewer individual connections survived connection-level FDR correction (see Supplementary Table S2). In a subsequent exploratory model including only the stroke patient group, time since stroke showed no significant associations with task-dependent connectivity.

To evaluate whether unequal group sizes influenced the findings, we repeated the second-level analyses five times using randomly selected subsamples of 18 S patients to match the 18 controls. Cluster-level significance recurred in every run, and the core midbrain–striatal effects (e.g., increased connectivity in stroke for SN–Putamen, SN–NAcc, NAcc–SN, NAcc l–FOrb l, and decreased connectivity in stroke for NAcc–VTA, FOrb–NAcc) reappeared consistently. Orbitofrontal and premotor connections showed greater variability, but remain directionally consistent. A detailed list of replicated connections across subsamples is provided in Supplementary Table S3a, and a summary of replication stability by cluster is shown in Supplementary Table S3b.

## 4 Discussion

This study aimed to elucidate the neural mechanisms underlying reward processing deficits after stroke and their implications for motor recovery. For this purpose, we investigated differences in task-related functional connectivity within the reward network during monetary reward processing in a motor skill learning task, comparing subacute stroke survivors to healthy controls. Motivation is critical for rehabilitation success, as motor learning can be exhausting, and feedback with rewards plays a fundamental role in sustaining engagement and facilitating recovery.

Behavioural analyses revealed that stroke survivors performed similarly to healthy controls in the motor skill learning task, with both groups demonstrating comparable learning trajectories, as indicated by the convergence of their performance curves. Specifically, movement accuracy (mean % in channel) and intrinsic motivation ratings did not differ significantly between groups. However, stroke patients showed significantly lower cognitive performance (MoCA) and higher depressive symptoms (BDI) compared to controls. They also exhibited slower movement times during the task, suggesting subtle motor differences despite equivalent accuracy. Overall, the observed trend of decreasing response times (i.e., faster performance) and increasing accuracy across blocks suggests motor learning in both patients and controls, indicating that differences in reward processing are not driven by gross motor deficits but by neural network alterations.

The primary focus of the study was on task-related functional connectivity within the reward network, assessed using generalized psychophysiological interactions (gPPI). This approach allowed us to capture dynamic interactions between key regions during reward processing, offering insights into network-level alterations post-stroke. Unlike regional activation analyses, gPPI reveals context-dependent changes in connectivity, making it ideal for examining reward-driven neural dynamics.

Our findings reveal significant region-specific changes in the functional connectivity of the reward circuit, rather than a uniform connectivity shift, highlighting the complexity of post-stroke neural adaptations. More precisely, subacute stroke survivors predominantly show increased functional connectivity involving the VTA, SN, NAcc, Putamen, FOrb, and precentral gyrus. Conversely, reduced functional connectivity was found specifically between VTA and the right NAcc. These changes were specific to the reward interval, underscoring their relevance to reward processing. Including antidepressant use as a covariate preserved the core pattern of connectivity alterations, although some individual connections were attenuated, indicating that the findings are not solely attributable to medication status. In contrast, controlling for MoCA further reduced the statistical significance of individual connections, which is expected given that MoCA is a broad screening tool and strongly correlated with group status in our sample. Importantly, in both sensitivity analyses the overall direction and pattern of effects were preserved, supporting the robustness of our main findings. Time since stroke showed no significant associations with task-dependent connectivity, however, our study might be underpowered to make conclusions about the impact on time since stroke on reward pathways connectivity post-stroke. The subgroup analyses with balanced groups (18 vs 18; Supplementary Table S2) reproduced the cluster-level effects and the core midbrain–striatal hyperconnectivity pattern, indicating that our main findings are not driven by group-size imbalance. Variability in some orbitofrontal–motor edges likely reflects sample- and/or power-sensitivity rather than inconsistency in direction.

Interestingly, the functional connectivity between the VTA and the putamen and striatum, reflecting the interplay between motor and reward networks and associated with dopaminergic input from the VTA facilitating motor learning (Hosp et al., 2011, Leemburg et al., 2018), was increased in stroke patients, which might influence reward-driven motor learning. Previous studies suggest that excessive connectivity may impair motor learning by engaging less efficient neural pathways, leading to reduced performance efficiency (Hummel & Cohen, 2005). However, hyperconnectivity within the reward network has been linked to both compensatory mechanisms and maladaptive changes (Hummel & Cohen, 2005). Indicative of compensatory mechanisms in response to network disruptions, observed hyperconnectivity pattern in stroke survivors during reward processing could reflect reorganization and plasticity (Aswendt & Hoehn, 2023). Bassett et al. (2011) demonstrated that higher network flexibility, potentially linked to hyperconnectivity, is associated with better learning outcomes, suggesting that reorganization within functional networks is a critical factor in adaptation.

In our study, stroke survivors performed similarly to healthy controls in the motor skill learning task, suggesting that our findings may point to adaptive compensation. In this context, the observed hyperconnectivity in reward and reward-motor pathways may enhance engagement in task execution. In line, Buma et al. (2013) explains our observation of hyperconnectivity due to an imbalance between Hebbian learning and homeostatic mechanisms. Here, a possible explanation is that overactivation serves as a compensatory response due to reduced sensitivity of target areas to dopaminergic input following stroke-related damage. In this case, hyperconnectivity might help maintain functional engagement despite neurodisruption.

Interestingly, the observed hyperconnectivity between the NAcc and other reward-related regions may reflect compensatory mechanisms supporting motivation and goal-directed behavior after stroke. Despite neurological impairment, patients reported similar levels of motivation for motor training as healthy controls. Increased connectivity could reflect heightened reward sensitivity, stronger reward cravings, and/or altered psychological motivation mechanisms. Such hyperconnectivity might therefore represent an adaptive neural response that supports motivation for motor learning—consistent with theories suggesting that altered reward sensitivity can influence participation in rehabilitation (Robinson and Jorge, 2016, Husain and Roiser, 2018). This interpretation aligns with recent evidence that reward system plasticity may contribute to recovery processes (Aswendt & Hoehn, 2023).

Interestingly, while reduced striatal activation during reward processing after stroke has previously been reported using the same dataset, even in patients without direct lesions to the NAcc (Widmer et al., 2019), other findings point to hyperconnectivity of this region in specific subgroups, such as individuals with depressive symptoms (Oestreich et al., 2022). This highlights a complex picture: altered connectivity within the reward network may reflect either compensatory adaptation or dysfunctional reorganization, depending on clinical presentation and context. As such, hyper- or hypoconnectivity of the NAcc cannot be universally interpreted as either adaptive or maladaptive without considering co-occurring symptoms like depression, lesion location, and behavioral outcomes such as motor learning.

In contrast, the observed hypoconnectivity between VTA and NAcc in stroke patients, might indicate reduced reward signal, potentially compensated by hyperconnectivity between other reward areas. Alternatively, it could reflect reduced dopaminergic transmission, leading to a more general reward deficiency syndrome. Notably, this apparent reduction in sensitivity might paradoxically coexist with the not preserved motivation for motor learning in these patients, suggesting that motivational engagement can be sustained despite altered reward signaling pathways.

Alternatively, reduced functional connectivity may be attributed to diaschisis-related network disruption, a phenomenon where a stroke lesion affects functionally connected but structurally intact brain regions. This occurs due to a loss of excitatory input from the damaged area, leading to temporary or prolonged functional suppression in remote regions (Buma et al., 2013). However, a classical diaschisis effect may be unlikely in this context, as the observed connectivity alterations appear to be more influenced by motivational and reward-driven neural changes rather than structural disconnection alone.

Stroke heterogeneity significantly affects recovery trajectories, with both lesion size and time since stroke playing a crucial role in determining whether connectivity alterations manifest as hyperconnectivity or hypoconnectivity. In our subacute stroke sample with smaller lesions, we observed hyperconnectivity, which, according to Aswendt & Hoehn (2023) is expected to normalize over time. In contrast, larger lesions, particularly in chronic stroke, are more often associated with persistent hypoconnectivity and poorer outcomes (Aswendt & Hoehn, 2023). The degree to which these connectivity patterns correspond to functional deficits and recovery potential remains an important area for further investigation. Identifying whether hyperconnectivity represents a transient phase of neural reorganization or a long-term maladaptive response could guide the development of targeted therapeutic interventions. Specifically, interventions that capitalize on reward-based neuroplasticity, such as incentive-driven rehabilitation strategies, dopaminergic pharmacological support or non-invasive brain stimulation targeting connectivity alterations, may enhance functional recovery by leveraging intact or compensatory reward network pathways. This aligns with the notion that increased reward-seeking behaviors in subacute stroke patients may be leveraged in therapy to enhance engagement and optimize recovery outcomes.

Interestingly, we found no significant differences in functional connectivity during the no-reward interval, suggesting that observed changes are specific to reward processing rather than a general connectivity increase. However, without a formal interaction effect, this interpretation remains tentative. Future studies should explore whether these changes in functional connectivity correspond to behavioral differences in motivation and reward sensitivity among stroke survivors with varied lesion locations and severities.

Our study highlights the need of investigating reward network connectivity changes post-stroke. Future research should integrate differences across stroke subtypes and motor learning trajectories to help refine personalized rehabilitation strategies. Understanding the trajectory of these connectivity changes may be important for developing interventions that optimize motivation, engagement, and functional recovery in stroke survivors. Insights from this research could inform rehabilitation programs that utilize reward mechanisms, such as performance-based feedback and tailored incentives, or non-invasive brain stimulation targeting disrupted reward connectivity, to enhance motivation, learning, and thereby functional recovery.

## 5 Limitations

This study has several limitations. First, our sample size (28 S patients, 18 controls) is modest for ROI-to-ROI analyses, and IMI/monetary value ratings were only collected in a subset of participants (n = 20 S; n = 9 controls). While this number is comparable to prior task-based fMRI studies in stroke, the limited sample reduces statistical power, particularly for brain–behavior associations, which must therefore be interpreted with caution. In addition, the relatively low subjective value of the monetary reward (mean ratings 2.25 and 2.83 on a 7-point scale) raises uncertainty as to whether participants experienced the reward as strongly motivating, which may have influenced reward-processing interpretations (Liu et al., 2020). Second, our sample was unbalanced in terms of handedness, which may have influenced motor outcomes, particularly in regions like the precentral gyrus (Steiner et al., 2022). Lastly, the control group was smaller than the stroke group, which may have reduced statistical power. Future studies should address these factors to improve result robustness.

## 6 Conclusions

Taken together, our findings provide important groundwork that stroke survivors show altered functional connectivity in the reward network during processing of monetary reward. The observed hyperconnectivity in reward-motor pathways may reflect compensatory mechanisms supporting motor recovery, while hypoconnectivity could indicate diaschisis-related network disruption. These connectivity changes highlight the dynamic nature of post-stroke neuroplasticity, where early-phase adaptations may either facilitate recovery or lead to maladaptive reorganization. Future research should clarify whether hyperconnectivity normalizes over time and explore its relationship with motor learning and rehabilitation outcomes. Understanding these mechanisms may be important for optimizing targeted interventions that harness reward-based neuroplasticity to enhance functional recovery.

## CRediT authorship contribution statement

**Yésica E. Martínez:** Writing – review & editing, Writing – original draft, Visualization, Software, Resources, Methodology, Investigation, Formal analysis, Data curation, Conceptualization. **Mario Widmer:** Writing – review & editing, Validation, Supervision, Resources, Project administration, Investigation, Data curation, Conceptualization. **Josua Zimmermann:** Writing – review & editing, Visualization, Validation, Supervision, Software, Methodology, Formal analysis, Conceptualization. **Josef G. Schönhammer:** Writing – review & editing, Validation, Supervision, Resources. **Andreas R. Luft:** Writing – review & editing, Supervision, Resources, Project administration, Methodology, Funding acquisition, Conceptualization. **Lutz Jäncke:** Writing – review & editing, Supervision, Resources, Project administration, Methodology, Funding acquisition, Conceptualization.

## Declaration of Competing Interest

The authors declare that they have no known competing financial interests or personal relationships that could have appeared to influence the work reported in this paper.

## Data Availability

The authors do not have permission to share data.
