# Supplementary material for: Brain reorganization: altered functional connectivity in reward network after stroke
Source: Neuroimage Clin. 2025 Nov 22;48:103914. doi: 10.1016/j.nicl.2025.103914 (PMC12685550; doi:10.1016/j.nicl.2025.103914)
Supplement: Supplementary Data 1 [file mmc1.docx]

| **Figure S1. Samples of VTA registration for stroke patients in MNI space.** |
| --- |
| **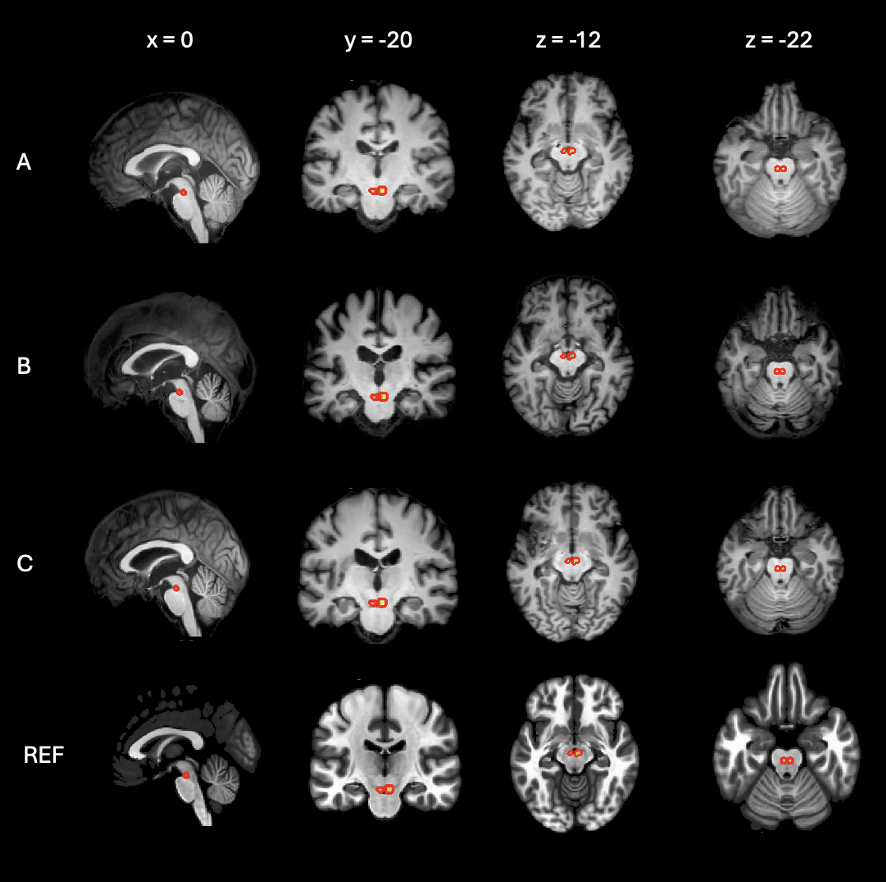** |
| *Note.* VTA ROI marked in red. A-C = stroke patients in MNI space. REF = Reference Standard Brain in MNI space. |

| **Figure S2. Samples of SN registration for stroke patients in MNI space.** |
| --- |
| 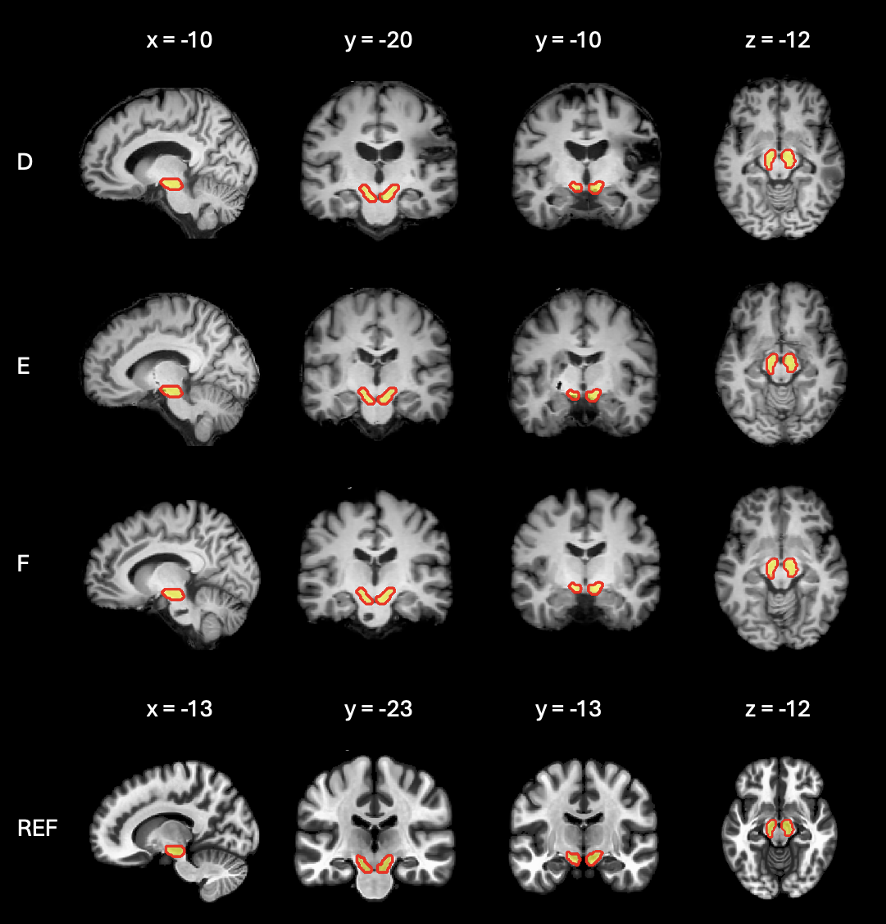 |
| *Note.* SN ROI marked in red. D-F = stroke patients in MNI space. REF = Reference Standard Brain in MNI space. |

| **Table S1. Sensitivity analysis of ROI-to-ROI connectivity during FB+Reward with age, sex, BDI, and antidepressant use as covariates.** | | | | | |
| --- | --- | --- | --- | --- | --- |
| **Connection** | ***β-weight*** | ***T*-Statistic** | ***p*-unc** | ***p*_FDR_** | **Comparison** |
| **Cluster 1/3** |  | **F(2,38) = 8.22** | **.001** | **.003** |  |
| Putamen l – SN | 2.01 | 4.01 | .000 | .003 | replicated |
| SN – Putamen l | 0.19 | 3.60 | .001 | .011 | replicated |
| SN – NAcc l | 0.32 | 3.31 | .002 | .013 | replicated |
| NAcc l – SN | 0.79 | 2.86 | .007 | .043 | replicated |
| NAcc l – VTA | 0.81 | 2.70 | .010 | .045 | replicated |
| Putamen l – VTA | 2.00 | 2.74 | .009 | .055 | replicated |
| Putamen l – PreCG l | 0.21 | 2.46 | .018 | .079 | new |
| SN – FOrb l | 0.14 | 2.15 | .038 | .123 | replicated |
| NAcc r – VTA | -0.76 | -2.60 | .013 | .170 | replicated |
| VTA – Putamen l | 0.10 | 2.43 | .020 | .246 | replicated |
| VTA – NAcc l | 0.22 | 2.15 | .038 | .246 | replicated |
| PreCG l – SN | 1.35 | 2.21 | .033 | .373 | new |
| Note. Note. *β*-weight represents the interaction between seed ROI activity and task interval (“FB+reward”), as modeled in gPPI analysis. Results demonstrate that the main findings replicated when age, sex, depressive symptom severity (BDI), and antidepressant use (yes/no) were included as covariates. Not all individual connections remained significant after FDR correction. This suggests that the results are partly attenuated but remain consistent in direction, supporting the robustness of the main effects. SN = substantia nigra; NAcc = nucleus accumbens; VTA = ventral tegmental area; FOrb = orbitofrontal cortex; PreCG = precentral gyrus. | | | | | |

| **Table S2. ROI-to-ROI functional connectivity results during the *FB+Reward* interval after including MoCA as an additional covariate in the gPPI analysis.** | | | | | |
| --- | --- | --- | --- | --- | --- |
| **Connection** | ***β-weight*** | ***T*-Statistic** | ***p*-unc** | ***p*FDR** | **Comparison** |
| Cluster 1/3 | - | F(2,37) = 5.74 | 0.007 | 0.020 |  |
| Putamen l – SN | 1.65 | 3.22 | 0.003 | 0.034 | replicated |
| SN – Putamen l | 0.16 | 2.94 | 0.006 | 0.064 | replicated |
| SN – NAcc l | 0.28 | 2.72 | 0.010 | 0.064 | replicated |
| NAcc l – VTA | 0.71 | 2.23 | 0.032 | 0.146 | replicated |
| NAcc l – SN | 0.64 | 2.20 | 0.034 | 0.146 | replicated |
| Putamen l – VTA | 1.64 | 2.13 | 0.040 | 0.174 | replicated |
| Note. *β*-weight represents the interaction between seed ROI activity and task interval (“FB+reward”), as modeled in gPPI analysis. When age, sex, depressive symptom severity (BDI), antidepressant use (yes/no), and MoCA scores were included as covariates, the overall pattern of connectivity findings was preserved, but fewer individual ROI-to-ROI connections remained significant after FDR correction. This indicates that the results are attenuated but directionally consistent, supporting the robustness of the main effects. SN = substantia nigra; NAcc = nucleus accumbens; VTA = ventral tegmental area. | | | | | |

| **Table S3a. ROI-to-ROI functional connectivity results during the *FB+Reward* interval after for balanced group comparison including five random groups of *n*=18 stroke patients.** | | | |
| --- | --- | --- | --- |
| **Random Group** | **Connection** | ***T*-Value** | ***p*_FDR_** |
| **Group 1** | **Cluster 1/8** | **F(2,30) = 14.35** | **.000** |
|  | Putamen l – VTA | 4.25 | .002 |
|  | SN – NAcc l | 3.86 | .005 |
|  | SN – Putamen l | 3.76 | .005 |
|  | NAcc l – SN | 3.17 | .022 |
|  | PreCG l – SN | 3.02 | .033 |
|  | SN – PreCG l | 2.58 | .048 |
|  | SN – PreCG r | 2.2 | .081 |
|  | PreCG r– SN | 2.87 | 0.095 |
|  | Cluster 2/8 | F(2,30) = 6.01 | 0.026 |
|  | VTA – Putamen l | 4.3 | 0.002 |
|  | Putamen l – VTA | 3.63 | 0.007 |
|  | VTA – NAcc l | 2.94 | 0.040 |
|  | NAcc l – VTA | 2.66 | 0.054 |
| **Group 2** | **Cluster 1/5** | **F(2,30) = 4.88** | **0.026** |
|  | NAcc l – SN | 5.13 | 0.000 |
|  | SN – NAcc l | 4.68 | 0.001 |
|  | Putamen l – SN | 4.38 | 0.002 |
|  | SN – Putamen l | 3.72 | 0.005 |
|  | SN – FOrb l | 2.42 | 0.093 |
|  | SN – Caudate l | 2.12 | 0.137 |
|  | PreCG l – SN | 2.08 | 0.242 |
|  | **Cluster 2/5** | **F(2,30) = 4.87** | **0.026** |
|  | Putamen l – VTA | 3.41 | 0.008 |
|  | NAcc l – VTA | 3.02 | 0.022 |
|  | VTA – Putamen l | 2.89 | 0.088 |
|  | VTA – NAcc l | 2.62 | 0.088 |
|  | NAcc r – VTA | -2.51 | 0.157 |
|  | **Cluster 3/5** | **F(2,30) = 4.78** | **0.026** |
|  | Putamen l – PreCG r | 3.49 | 0.008 |
|  | NAcc l – FOrb l | 3.07 | 0.022 |
|  | FOrb r – NAcc l | -2.94 | 0.051 |
|  | FOrb r – PreCG l | 2.84 | 0.051 |
|  | Caudate r – NAcc l | 2.97 | 0.075 |
|  | NAcc l – FOrb r | -2.36 | 0.081 |
|  | Putamen l – FOrb l | 2.11 | 0.123 |
|  | Putamen l – NAcc l | 2.07 | 0.123 |
|  | NAcc r – FOrb l | 2.27 | 0.157 |
|  | NAcc r – PreCG l | -2.19 | 0.157 |
|  | FOrb l – FOrb r | -2.41 | 0.174 |
|  | FOrb l – NAcc l | 2.33 | 0.174 |
|  | PreCG l – FOrb r | 2.26 | 0.242 |
|  | Pallidum r – PreCG l | -2.23 | 0.434 |
|  | PreCG r – Putamen l | 2.17 | 0.457 |
| **Group 3** | **Cluster 1/8** | **F(2,30) = 20.34** | **0.000** |
|  | Putamen l – SN | 5.64 | 0.000 |
|  | SN – NAcc l | 5.07 | 0.000 |
|  | SN – Putamen l | 4.92 | 0.000 |
|  | NAcc l – SN | 4.41 | 0.001 |
|  | SN – Pallidum r | 2.08 | 0.088 |
|  | PreCG r – SN | 2.89 | 0.092 |
|  | Pallidum r – SN | 2.52 | 0.112 |
|  | **Cluster 2/8** | **F(2,30) = 18.97** | **0.000** |
|  | VTA – Putamen l | 5.5 | 0.000 |
|  | Putamen l – VTA | 5.22 | 0.000 |
|  | VTA – NAcc l | 3.4 | 0.012 |
|  | NAcc l – VTA | 2.86 | 0.033 |
|  | NAcc r – VTA | -2.73 | 0.068 |
|  | **Cluster 3/8** | **F(2,30) = 6.58** | **0.011** |
|  | FOrb l – FOrb r | -4.35 | 0.002 |
|  | FOrb r – Pallidum l | 2.13 | 0.139 |
|  | **Cluster 4/8** | **F(2,30) = 5.67** | **0.014** |
|  | VTA – Pallidum l | 3.15 | 0.016 |
|  | Pallidum l – VTA | 3.45 | 0.021 |
|  | VTA – Caudate l | 2.41 | 0.071 |
|  | **Cluster 5/8** | **F(2,30) = 5.53** | **0.014** |
|  | SN – FOrb l | 3.2 | 0.014 |
|  | SN – Caudate l | 2.95 | 0.019 |
|  | SN – Caudate r | 2.23 | 0.086 |
|  | SN – Pallidum l | 2.07 | 0.088 |
| **Group 4** | **Cluster 1/3** | **F(2,30) = 11.84** | **0.000** |
|  | SN – Putamen l | 3.48 | 0.020 |
|  | Putamen l – SN | 3.32 | 0.030 |
|  | NAcc r – SN | -3.09 | 0.037 |
|  | NAcc r – VTA | -2.97 | 0.037 |
|  | SN – NAcc l | 2.73 | 0.067 |
|  | SN – NAcc r | -2.32 | 0.118 |
|  | NAcc l – SN | 2.46 | 0.138 |
|  | NAcc l – VTA | 2.23 | 0.138 |
|  | VTA – NAcc l | 2.08 | 0.406 |
| **Group 5** | **Cluster 1/8** | **F(2,30) = 10.67** | **0.003** |
|  | SN – NAcc l | 4.12 | 0.002 |
|  | SN – Putamen l | 3.85 | 0.002 |
|  | Putamen l – SN | 3.82 | 0.008 |
|  | NAcc rl– SN | 3.26 | 0.018 |
|  | PreCG l – SN | 2.46 | 0.127 |
|  | PreCG r – SN | 2.51 | 0.228 |
|  | **Cluster 2/8** | **F(2,30) = 8.70** | **0.004** |
|  | FOrb r – PreCG l | 4.45 | 0.001 |
|  | NAcc l – FOrb l | 3.96 | 0.005 |
|  | PreCG l – FOrb l | 3.95 | 0.006 |
|  | Putamen l – FOrb l | 2.72 | 0.046 |
|  | FOrb r – PreCG r | 2.77 | 0.061 |
|  | FOrb l – NAcc l | 3.03 | 0.064 |
|  | FOrb r – NAcc l | -2.5 | 0.078 |
|  | Putamen l – Pallidum l | 2.12 | 0.092 |
|  | NAcc l – FOrb r | -2.15 | 0.128 |
|  | NAcc r – PreCG l | -2.2 | 0.186 |
|  | Pallidum l – PreCG l | 2.04 | 0.323 |
| *Note.* The table reports the detailed connectivity results from five robustness checks with equalized group sizes. For each random subsample, significant clusters and individual ROI-to-ROI connections are listed with corresponding test statistics and FDR-corrected p-values. These analyses demonstrate that the main midbrain–striatal effects (e.g., Putamen–SN, SN–NAcc, SN–Putamen, NAcc–SN) were consistently observed across subsamples, while orbitofrontal and premotor connections showed greater variability, likely reflecting reduced statistical power in smaller samples. FC = functional connectivity; SN = substantia nigra; NAcc = nucleus accumbens; VTA = ventral tegmental area; Forb = orbitofrontal cortex; PreCG = precentral gyrus. | | | |

| **Table S3b. Robustness of ROI-to-ROI functional connectivity results during the FB+Reward interval across five balanced subsamples (18 stroke vs. 18 control patients).** | | | | | | |
| --- | --- | --- | --- | --- | --- | --- |
| **Cluster** | **Connection** | ***β-weight*** | **FC in Stroke** | **Replication in n/5** | **Direction confirmed** |  |
| **1** | Putamen l – SN | 2.05 | Higher | 4 | yes |  |
|  | SN – NAcc l | .38 | Higher | 5 | yes |  |
|  | SN – Putamen l | .20 | Higher | 5 | yes |  |
|  | NAcc l – SN | .90 | Higher | 3 | yes |  |
|  | Putamen l – VTA | 2.07 | Higher | 4 | yes |  |
|  | NAcc r – VTA | -.90 | Lower | 3 | yes |  |
|  | SN – FOrb l | .16 | Higher | 2 | yes |  |
|  | NAcc l – VTA | .71 | Higher | 4 | yes |  |
|  | VTA – Putamen l | .11 | Higher | 3 | yes |  |
|  | VTA – NAcc l | .24 | Higher | 4 | yes |  |
|  | Precentral gyrus r – SN | 1.23 | Higher | 2 | yes |  |
|  | Pallidum l – VTA | 1.00 | Higher | 1 | yes |  |
| **2** | NAcc l – FOrb l | .28 | Higher | 1 | yes |  |
|  | Putamen l – Precentral gyrus r | .21 | Higher | 1 | yes |  |
|  | FOrb l – FOrb r | -.38 | Lower | 2 | yes |  |
|  | Putamen l – FOrb l | .44 | Higher | 0 | - |  |
|  | FOrb r – Precentral gyrus l | .38 | Higher | 2 | yes |  |
|  | FOrb r – NAcc l | -.71 | Lower | 2 | yes |  |
|  | FOrb l – NAcc l | .80 | Higher | 2 | yes |  |
|  | FOrb l – Caudate r | .47 | Higher | 0 | - |  |
|  | NAcc r **–** FOrb l | .20 | Higher | 0 | - |  |
|  | Precentral gyrus r **–** Putamen l | .49 | Higher | 1 | yes |  |
|  | Caudate r **–** NAcc l | .42 | Higher | 1 | yes |  |
|  | Caudate r **–** Caudate l | .27 | Higher | 0 | - |  |
|  | Precentral gyrus l **–** FOrb r | .45 | Higher | 1 | yes |  |
|  | Pallidum r **–** Precentral gyrus l | **-**.19 | Lower | 0 | - |  |
| *Note.* The table summarizes whether each main connection identified in the full-sample analysis was replicated across five random balanced subsamples. “Replication in n/5” indicates in how many of the five subsamples the effect was significant at cluster-level FDR. “Direction confirmed” indicates whether the direction of the effect (higher/lower in stroke vs. control) matched the original result in all subsamples. Connections are presented separately for Cluster 1 (above the line) and Cluster 2 (below the line), based on the main analysis (see Table 4). The order of connections corresponds to Table 4 to facilitate direct comparison. Connections in Cluster 1, primarily within the midbrain–striatal circuitry, showed higher replication rates across subsamples and appear more robust, whereas connections in Cluster 2 were less consistent and more sample-sensitive. FC = functional connectivity; SN = substantia nigra; NAcc = nucleus accumbens; VTA = ventral tegmental area; Forb = orbitofrontal cortex; PreCG = precentral gyrus. | | | | | | |
